# Immediate Psychosocial Impact on Healthcare Workers During COVID-19 Pandemic in China: A Systematic Review and Meta-Analysis

**DOI:** 10.3389/fpsyg.2021.645460

**Published:** 2021-05-28

**Authors:** Fei Dong, Hong-liang Liu, Ming Yang, Chun-li Lu, Ning Dai, Ying Zhang, Nicola Robinson, Jian-ping Liu

**Affiliations:** ^1^Center for Evidence-Based Chinese Medicine, Beijing University of Chinese Medicine, Beijing, China; ^2^School of Traditional Chinese Medicine, Beijing University of Chinese Medicine, Beijing, China; ^3^Gastroenterology Department, Guang'anmen Hospital, China Academy of Chinese Medicine Sciences, Beijing, China; ^4^Institute of Health and Social Care, London South Bank University, London, United Kingdom; ^5^Institute for Excellence in Evidence-Based Chinese Medicine, Beijing University of Chinese Medicine, Beijing, China

**Keywords:** anxiety, COVID-19, depression, meta-analysis, psychosocial impact, systematic review

## Abstract

**Objectives:** The corona virus disease-2019 (COVID-19) pandemic spread globally, and we aimed to investigate the psychosocial impact on healthcare workers (HWs) in China during the pandemic.

**Methods:** In this systematic review and meta-analysis, we searched seven electronic databases for cross-sectional studies on psychosocial impact on HWs in relation to COVID-19 from January 1, 2020 to October 7, 2020. We included primary studies involving Chinese HWs during the pandemic, and data were extracted from the published articles. Our primary outcome was prevalence of anxiety, depression, and stress disorders. We pooled prevalence value with their 95% confidence interval using random effect models and assessed study quality on the basis of an 11-item checklist recommended by the Agency for Healthcare Research and Quality. The study protocol was registered in PROSPERO (CRD42020195843).

**Results:** We identified 25 articles comprising a total of 30,841 completed questionnaires and 22 studies for meta-analysis. The prevalence of anxiety, depression, and stress disorders was 34.4% (29.5–39.4%), 31.1% (24.5–37.7%), and 29.1% (24.3–33.8%) for HWs. The pooled prevalence of anxiety disorders for HWs from late January to early February was 46.4% (42.9–49.9%), significantly higher than those in mid-term February (28.0%, 23.9–32.1%) and after late February (27.6%, 16.0–39.2%). The pooled prevalence of depression disorders for HWs from late January to early February was 46.5% (38.8–54.2%), significantly higher than those in mid-term February (27.1%, 19.8–34.5%) and after late February (32.9%, 16.2–49.5%). HWs working in Hubei Province had a higher prevalence of anxiety (37.9 vs. 30.8%) and a lower prevalence of depression (27.5 vs. 34.7%) than those working in other regions. Nurses had a higher prevalence of anxiety (44.1 vs. 29.0%) and depression (34.1 vs. 29.2%) than other HWs.

**Conclusions:** About one-third of HWs in China suffered anxiety, depression, and stress at the early epidemic of COVID-19. HWs in Hubei Province, especially nurses, had a higher prevalence of psychological disorders. During the pandemic, a negative psychological state may persist in a proportion of Chinese HWs, fluctuating with the control of the pandemic. The long-term impact should continue to be observed. Attention should be paid to HWs for their psychological impact due to the pandemic.

**Systematic Review Registration:** The study protocol was registered with PROSPERO (CRD42020195843).

## Introduction

The corona virus disease-2019 (COVID-19) pandemic has swept across the world. Over the course of the pandemic, many countries and regions have adopted preventive measures, such as lockdown, to regulate movement and workplaces (Brooks et al., 2020). Scholars across the globe have likewise highlighted the need to better understand psychosocial problems caused by the pandemic (Duan and Zhu, 2020; Holmes et al., 2020; Liu S. et al., 2020; Shigemura et al., 2020). Indeed there is a need for research that evaluates the mental health of healthcare workers (HWs) who are caring for patients during a viral outbreak and the potential psychosocial interventions (Kisely et al., 2020).

The COVID-19 pandemic has had a devastating effect on people's physical and mental health (Galea et al., 2020; Moreno et al., 2020). Published research on COVID-19 has identified the negative effects the pandemic has had on the mental health of different populations, causing conditions such as anxiety, depression, and stress (Cai W. et al., 2020; Liu Q. et al., 2020; Ni et al., 2020; Park and Park, 2020). As information on COVID-19 becomes available to the public, psychological distress resulting from repeated media exposure to the outbreak has emerged (Garfin et al., 2020). According to existing research, the more negative information one receives, the more inclined one is to feel stressed (Garfin et al., 2020). The general population has been quarantined in their homes; HWs and frontline workers involved in epidemic prevention must deal with the physical hardship of long working hours and the unavoidable mental stress caused by negative information from the media (Cao et al., 2020; Gao et al., 2020). Due to the unpredictability of the disease and the necessary isolation of patients, those who have been diagnosed with COVID-19 and people who have been medically quarantined were very likely to be anxious and depressed (Bo et al., 2020; Wang C. et al., 2020). According to existing research, patients with cancer and other underlying diseases exhibited increased psychological discomfort during the COVID-19 pandemic and received different levels of mental health services (Naqos and Khouchani, 2020; Wang Y. et al., 2020). Therefore, the COVID-19 pandemic has also posed high risks for other patients.

Healthcare workers around the globe have been involved in outbreak control and the treatment of patients. Compared with other groups, HWs have had to work longer hours under intense pressure. Increasing confirmed and suspected cases, heavy workloads, shortages of personal protective equipment, information overload, demand for specific drugs, and a lack of public support are all possible contributors to the psychological burdens faced by HWs (Fan et al., 2020; Galehdar et al., 2020; Liu Q. et al., 2020). For example, the Chinese government sent more than 42,000 HWs to treat patients in the affected areas of Hubei Province (Yao and Xu, 2020). Among these works, some lacked experience and expertise in infectious diseases before the COVID-19 pandemic and therefore faced additional hardships. However, even those medical workers who were not dispatched to remote locations and instead have worked at local hospitals have also faced significantly increased workloads and great challenges (Lai et al., 2020).

Soon following the outbreak of COVID-19, researchers carried out cross-sectional studies to analyze the psychosocial problems faced by HWs; researchers have also undertaken regular systematic reviews. Some reviews (Kisely et al., 2020; Krishnamoorthy et al., 2020; Luo et al., 2020; Pan et al., 2020; Pappa et al., 2020; Salazar De Pablo et al., 2020; Serrano-Ripoll et al., 2020; Da and Neto, 2021) have included evaluations and meta-analyses of the psychosocial problems faced by HWs during the COVID-19 pandemic. According to the evidence presented by researchers, a substantial proportion of HWs have experienced anxiety, depression, stress, sleep disorders, and other mental health problems during the outbreak. These findings emphasize a working mechanism of reduced risk to mental health and timely adjustments to psychological interventions in the context of the COVID-19 pandemic. However, these reviews pertain to journal articles published between March 2020 and May 2020 and only included cross-sectional studies published during the early months of the epidemic. It is likely that the COVID-19 pandemic will continue for quite some time. Therefore, it is necessary to update research evidence in a timely manner and track the changes in the mental health of HWs. Some studies provided only a qualitative description of existing literature as part of their narrative evaluations or general reviews (Barello et al., 2020; Bohlken et al., 2020; Braquehais et al., 2020; Chow et al., 2020; Fu et al., 2020; Heath et al., 2020; Magill et al., 2020; Muller et al., 2020; Paiano et al., 2020; Preti et al., 2020; Shaukat et al., 2020; Shreffler et al., 2020; Stuijfzand et al., 2020). Moreover, these studies neglected to include a quantitative evaluation of the strength, quality, and consistency of existing evidence. According to qualitative analysis, factors such as sex, age, specific job role, and experience in communicable disease control have influenced the mental health of HWs during the pandemic. Moreover, in different social and cultural contexts, people take different measures to deal with stress; this also applies to doctors, nurses, and other HWs (Cabarkapa et al., 2020). One study (Thombs et al., 2020) employed a relatively novel method of systematic review: living systematic review. Indeed among studies on the COVID-19 pandemic, providing dynamic updates of specific questions is the most desirable method. However, in September 2020, the research group announced that it would stop updating research of “factors associated with levels or changes in symptoms” because of the rapid growth in the number of low-quality cross-sectional studies and an inadequate number of group members. Therefore, living systematic evaluation will no longer provide the latest evidence regarding changes in the psychological health of different populations in this review.

The characteristics of COVID-19 outbreaks and disease prevention and control measures vary drastically across different nations and are affected by local cultural conditions. Therefore, we believe that research on the mental health of HWs should be specific to each area in order to produce more targeted interventions. Moreover, with changes in the situation of disease prevention and the deepening of our understanding of COVID-19, it is necessary to determine whether the mental health of HWs will change accordingly. This is a question worth exploring. Nevertheless, no existing research has addressed the mental health of Chinese HWs during the epidemic.

As a result, we conducted a systematic review and meta-analysis to explore changes in the mental health of health workers, examining the prevalence of anxiety, depression, and stress disorders among this group. Compared with previous research, this research evaluates not only the mental health of Chinese HWs but also the prevalence of psychological problems in different stages and differences across different levels of involvement in disease prevention and different posts.

## Methods

### Search Strategy and Selection Criteria

We did this systematic review and meta-analysis following the Preferred Reporting Items for Systematic Reviews and Meta-analyses guidelines (known as PRISMA; Supplementary Material, pp. 2–4) and Meta-analyses of Observational Studies in Epidemiology (known as MOOSE; Supplementary Material, pp. 5 and 6) guidelines.

In this systematic review, the study population was Chinese HWs and the main outcome of the prevalence of abnormal psychosocial state and its change. We found that there were a number of cross-sectional surveys, and the quality was low in a previous literature review; therefore, this study reviewed the literature in observational studies, such as cross-sectional and longitudinal studies, not involving the study of intervention measures, but the sample must be able to represent the overall population.

Based on comprehensive searching in seven electronic databases, including PubMed, Embase, PsycINFO, Wanfang Data, Chongqing VIP, Sinomed, and Chinese National Knowledge Infrastructure databases, we established the COVID-19 Mental Health Database. The search strategies of all databases could be seen in the Supplementary Material, pp 7–9. The limited publication languages are English or Chinese, published between January 1, 2020 and October 7, 2020. We selected studies from the COVID-19 Mental Health Database according to inclusion and exclusion criteria.

#### Inclusion Criteria

♦ Date of the studies: any study carried out between January 1, 2020 and October 7, 2020.
♦ Subjects: Chinese HWs under the COVID-19 epidemic, regardless of age and gender. There was no restriction on ethnicity.
♦ Study design: observational studies, such as cross-sectional sampling survey and longitudinal study.
♦ Articles that have been officially published or published online, conference articles, or other gray literature.
♦ Outcome indicators: the prevalence rate of mental health and psychological disorders, such as depression, anxiety, and stress.

#### Exclusion Criteria

♦ Duplicated research.
♦ Literature without data required for the research from the original text.
♦ No response rate or sampling could not infer the overall populations (if convenience sampling method only was used).
♦ Survey tools: self-designed questionnaire.

In order to ensure the comprehensiveness of published data, this study intended to extend and search the reference list of literature.

The study protocol was registered with PROSPERO (CRD42020195843) before the systematic review was done. Due to the increasing number of cross-sectional studies, the studies which related to this systematic protocol involving different populations had to be split into different research papers. This paper discussed the psychosocial impact on HWs.

### Data Analysis

Two authors (FD and HL) independently selected the literature, extracted data, and cross-checked for duplications. If two or more articles came from the same research, the one with the most complete data and the most detailed report was selected. Any differences were adjudicated through discussion or consultation with a third member of the research team. Documents were selected by reading the title and abstract first and then the full text in order to determine whether to include them in the study. If necessary, authors of the original study were contacted by email and telephone if there was any uncertainty or if important information was missing for the study. The data extraction form included the following: (1) the first author, research topic, and publication year; (2) characteristics of the studies: population category, research location, number of participants, gender, and departments; (3) key elements to assess bias and risk: research method, sampling method, survey form, survey tools, number of valid questionnaires, and survey time; (4) outcome indicators and outcome measurement data: number of reports of anxiety (mild, moderate, and severe), depression (mild, moderate, and severe), stress disorder, and scores on the SCL-90 scale. Findings of any other related psychosocial problems were also recorded. Frontline HWs were defined as doctors or nurses from departments of infectious diseases, emergency medicine, fever clinics, and intensive care units and included technicians from radiology and laboratory medicine and HWs working in infection prevention and those that directly faced and treated confirmed or suspected COVID-19 patients.

The methodological quality of the studies included was assessed using an 11-item checklist which was recommended by the Agency for Healthcare Research and Quality (Rostom et al., 2004). An item would be scored “0” if it was answered “NO” or “UNCLEAR”; if it was answered “YES,” then the item score was “1.” The study quality was assessed as follows: low quality = 0–3, moderate quality = 4–7, and high quality = 8–11. Two authors (FD and HL) evaluated the methodological quality and cross-checked the results independently. Any differences were adjudicated through discussion or consultation with a third member of the research team.

Prevalence was tabulated as the number of cases detected divided by the sample size, along with standard errors, and all estimates were expressed as a percentage of the population. There were two conditions to meet in order for a given study to be included in the meta-analysis: (1) it should have used a generally recognized scale to determine the outcome rather than a self-designed questionnaire and (2) there should be accurate reporting of the number of people who have the outcome or the scores of all dimensions of specific scales. The inverse variance method by DerSimonian and Laird (adjusted) (DerSimonian and Laird, 2015) was used to calculate pooled prevalence and 95% confidence intervals for prevalence rates and estimate values. Heterogeneity between studies was tested using the *I*^2^ statistic. Forest plots were used to display the results graphically. Due to the need for appropriate interventions for moderate and severe anxiety or depressive disorders, meta-analysis was conducted according to two groups (mild, moderate, and severe). In addition, a sensitivity analysis was performed to test the influence of possible outliers. Similarly, the presence of publication bias was tested using Begg's test and Egger's test. *P* < 0.05 will be considered statistically significant. The meta-analysis will be performed using Stata v 16.0.

## Results

### Search Results

**Figure 1:**
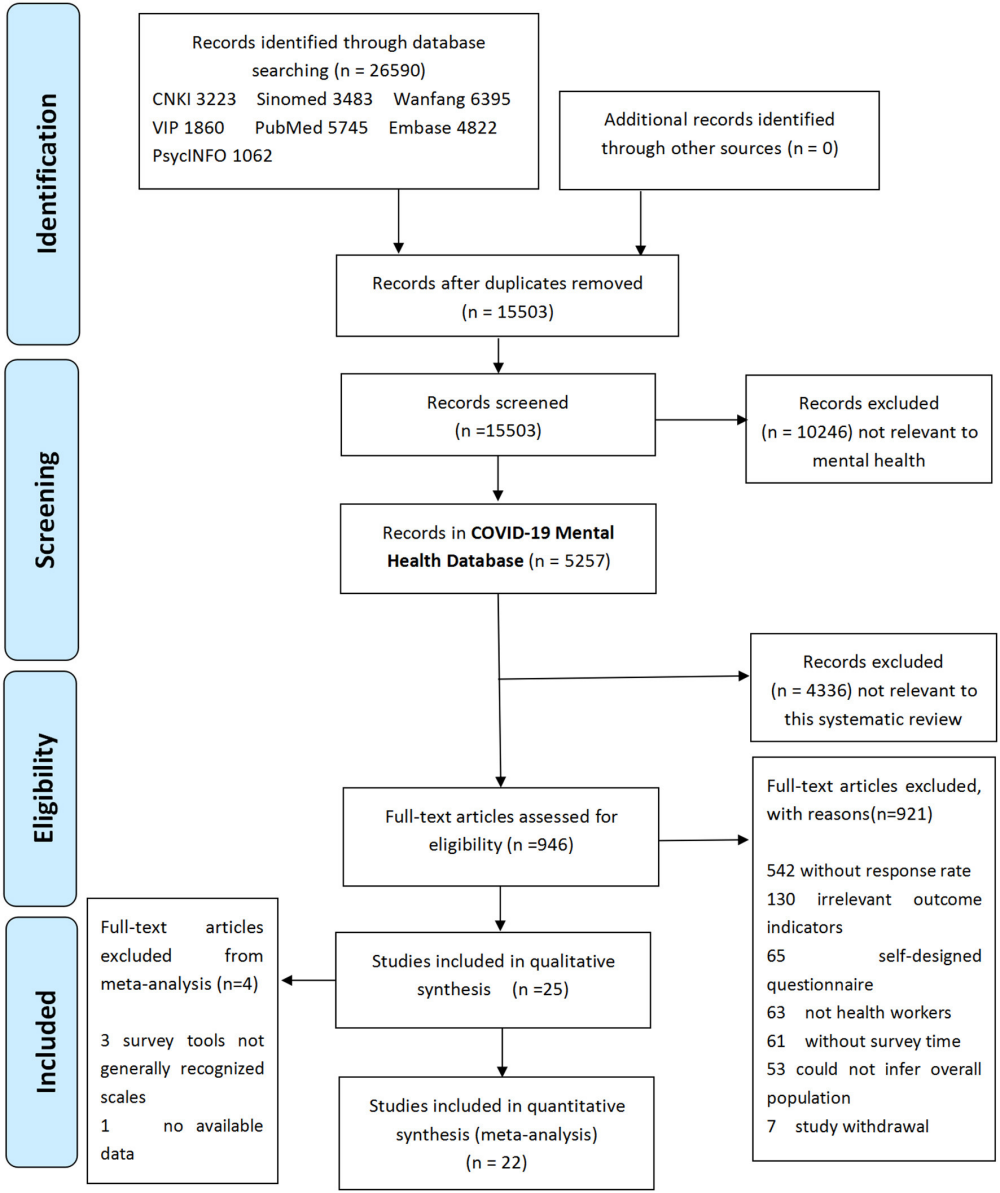
Flow diagram of the selection of studies for the systematic review and meta-analysis.

A total of 26,590 pieces of literature were obtained through preliminary COVID-19 mental health database screening, and 15,503 were left after elimination of duplicates. A total of 5,257 articles were obtained in COVID-19 Mental Health Database (Dynamic Version, date 2020-11-10) after eliminating obvious irrelevant review topics and abstracts. A total of 946 full-text articles from the COVID-19 Mental Health Database were reviewed and classified, and 25 studies were finally included in this review (see Figure 1).

### Study Characteristics

In this review, 25 studies were identified through the search as eligible for inclusion. Table 1 provides the characteristics of literature included in the systematic review. Of the 25 articles, 10 (Cai Z. et al., 2020; Hu et al., 2020; Lai et al., 2020; Li G. et al., 2020; Si et al., 2020; Tian et al., 2020; Wang S. et al., 2020; Xu X. et al., 2020; Yang et al., 2020; Zhu et al., 2020) were published in English, and 15 (Chen X. et al., 2020; Chen Y. et al., 2020; Gu et al., 2020; Guo et al., 2020; Huang et al., 2020; Li et al., 2020a,b; Liu et al., 2020a,b; Nong et al., 2020; Sun and Yu, 2020; Wei et al., 2020; Wu and Ding, 2020; Xu J. et al., 2020; Zheng and Zhu, 2020) were published in Chinese. Fourteen studies highlighted their study population as frontline HWs, six studies included only nurses, and one study surveyed residents. Fifteen study sites were located in Hubei Province, China. One study (Cai Z. et al., 2020) proceeds as a longitudinal study and contained two cross-sectional surveys. Twenty-five studies comprised 30,841 completed questionnaires. Online questionnaires were used in 18 studies (72.0%), which were sent out by researchers through *WeChat* or *Wenjuanxing* apps (similar to SurveyMonkey). Twenty-three studies (92.0%) used cluster sampling method. All the included cross-sectional studies were proceeded from the end of January to April 2020. The survey time of 19 studies (76.0%) was from the end of January 2020 to February 2020.

**Table 1:** Characteristics of the included studies reporting on healthcare workers' psychosocial status during the COVID-19 pandemic.

**No**.	**First author**	**Population**	**Medical facilities**	**Study location**	**Number of participants (*n*)**	**Response rate (%)**	**Sampling method**	**Survey form**	**Survey tools for anxiety, depression, stress**	**Completed questionnaire(*n*)**	**Completeness rate of data collection (%)**	**Gender (*****n*****)**		**Survey time**
												**Male**	**Female**	
1	Zi-feng Li	Frontline nurses	A designated hospital for COVID-19	Yichang City, Hubei Province	773	100	Cluster sampling	Online questionnaire survey through *Wenjuanxing* APP	SAS	773	100	95	678	02/2020
2	Qiu-xiang Nong	Psychiatric nurses	Two psychiatric hospitals	Guangxi Province	92	100	Randomized cluster sampling	Questionnaire survey	SCL-90, SDS, SAS	92	100	29	63	31/01/2020–03/02/2020
3	Yu-ning Chen	Residents	The First Affiliated Hospital,Zhejiang University School of Medicine	Hangzhou City, Zhejiang Province	712	71.27	Cluster sampling	Questionnaire survey	PHQ-9, GAD-7	711	99.86	315	396	03/02/2020–16/02/2020
4	Na Zheng^a^	Non-frontline nurses	Tongji Hospital, Tongji Medical College	Wuhan City, Hubei Province	118	100	Cluster sampling	Questionnaire survey	SCL-90	118	100	2	116	20/01/2020–05/02/2020
5	Xiao-wen Gu	Frontline nurses	The Third People's Hospital of Shenzhen	Shenzhen City, Guangdong Province	564	100	Convenience and cluster sampling	Questionnaire survey	SAS, SDS	564	100	NR	NR	01/2020–02/2020
6	Wei Wei^a^	Nurses in operating room	The First Affiliated Hospital of Zhengzhou University	Zhengzhou City, Henan Province	401	96.39	Cluster sampling	Online questionnaire survey through *Wenjuanxing* APP	PQEPHE	401	100	66	335	03/02/2020–08/02/2020
7	Xiao-lei Liu	Nurses	Chinese PLA General Hospital	Beijing City	1,097	100	Cluster sampling	Online questionnaire survey	PHQ-9, GAD-7, SRQ-20	1,097	100	19	1,078	01/02/2020–18/02/2020
8	Jia-qi Xu	Nurses	Tongji Hospital, Tongji Medical College	Wuhan City, Hubei Province	136	100	Cluster sampling	Online questionnaire survey through *Wenjuanxing* APP	PHQ-9, GAD-7, Fear NRS	136	100	0	136	29/01/2020–31/01/2020
9	Ji-zheng Huang	Frontline HWs	A designated hospital for COVID-19	Fuyang City, Henan Province	230	93.50	Cluster sampling	Online questionnaire survey through *Wenjuanxing* APP	SAS, PTSD-SS	230	100	43	187	07/02/2020–14/02/2020
10	Xia Chen	Frontline HWs	National Aid Hubei Medical Team from Xinjiang Uygur Autonomous Region	Hubei Province	386	100	Cluster sampling	Online questionnaire survey	SAS	361	93.52	19	342	28/01/2020–29/02/2020
11	Di Wu	Frontline HWs	National Aid Hubei Medical Team from Henan Province	Hubei Province	480	62.70	Cluster sampling	Online questionnaire survey	SQR	620	100	241	379	29/02/2020–01/03/2020
12	Zhen-xiao Sun	HWs in psychiatry department	Mental Health Center of Linyi City	Linyi City, Shandong Province	121	97.58	Random sampling	Questionnaire survey	PHQ-9,GAD-7	121	100	40	81	05/03/2020
13	Zheng Li	Frontline nurses	National Aid Hubei Medical Team from Shanxi Province	Hubei Province	133	100	Cluster sampling	Online questionnaire survey	DASS-21	130	97.74	9	121	06/02/2020–20/02/2020
14	Xiang-lai Liu	Frontline HWs	National Aid Hubei Medical Team from Hainan Province	Hubei Province	221	90.95	Cluster sampling	Online questionnaire survey through *Wenjuanxing* APP	SAS, SDS, SRQ-20, PCL-C	221	100	73	148	17/03/2020
15	Zhong-wei Guo	Frontline HWs	National Aid Hubei Medical Team from Zhejiang Province	Hubei Province	130	86.67	Cluster sampling	Online questionnaire survey through *Wenjuanxing* APP	PHQ-9, GAD-7	130	100	45	85	10/02/2020–12/02/2020
16	Xiao-ming Xu	Frontline HWs	Designated hospitals for COVID-19	Chongqing City	8,817	90.62	Cluster sampling	Online questionnaire survey through *Chongyixinli* platform	PHQ-9, GAD-7	8,817	100	1,943	6,874	14/02/2020–23/02/2020
17	S. Wang	HWs	Children's Healthcare Centre of Renmin Hospital of Wuhan University	Wuhan City, Hubei Province	129	52.44	Cluster sampling	Questionnaire survey	SAS, SDS	123	95.35	12	111	30/01/2020–07/02/2020
18	Ming-yu Si	HWs	Hospitals of seven Geographical regions in China	Seven provinces in mainland China	863	75.57	Purpose sampling	Online questionnaire survey	IES-6, DASS-21	863	100	253	610	23/02/2020–05/03/2020
19	Juan Yang	HWs	Five designated hospitals for COVID-19	Chongqing City	456	91.2	Cluster and random sampling	Online questionnaire survey	PHQ-9, GAD-7, IES-R	456	100	134	222	01/02/2020–14/02/2020
20	Zhong-xiang Cai	Nurses	Renmin Hospital of Wuhan University	Wuhan City, Hubei Province	709	72.94	Cluster sampling	Online questionnaire survey through *Wechat* APP	PHQ-9, GAD-7, IES-R	709	100	25	684	28/01/2020–02/02/2020
		Nurses	Renmin Hospital of Wuhan University	Wuhan City, Hubei Province	621	63.89	Cluster sampling	Online questionnaire survey through *Wechat* APP	PHQ-9, GAD-7, IES-R	621	100	16	605	26/02/2020–28/02/2020
21	Teng-fei Tian	Frontline HWs	Beijing Xiaotangshan Hospital	Beijing City	845	79.94	Cluster sampling	Online questionnaire survey through *Wenjuanxing* APP	PSS-10, PHQ-9, GAD-7, ISI	845	100	131	714	06/04/2020–10/04/2020
22	De-ying Hu^a^	Frontline nurses	Wuhan Union Hospital and Huo Shen Shan Hospital	Wuhan City, Hubei Province	2,110	81.15	Cluster sampling	Online questionnaire survey through *Wenjuanxing* APP	SAS, SDS	2,014	95.45	260	1,754	13/02/2020–24/02/2020
23	Zhou Zhu	Frontline HWs	Tongji Hospital, Tongji Medical College	Wuhan City, Hubei Province	5,281	80.40	Cluster sampling	Online questionnaire survey through *Wenjuanxing* APP	PHQ-9, GAD-7, IES-R	5,062	95.85	758	4,304	08/02/2020–10/02/2020
24	Jian-bo Lai	Frontline HWs	34 hospitals equipped with fever clinics or wards for COVID-19	20 hospitals in Wuhan City, 7 hospitals in other regions of Hubei province, and 7 hospitals from 7 otherprovinces with a high incidence of COVID-19	1,257	68.69	Region-stratified and 2-stage cluster sampling	Questionnaire survey	PHQ-9, GAD-7, IES-R	1,257	100	293	964	29/01/2020–03/02/2020
25	Guo Li	Female frontline HWs	Tongji Hospital, Tongji Medical College	Wuhan City, Hubei Province	4,369	82.17	Cluster sampling	Online questionnaire survey through *Wenjuanxing* APP	PHQ-9, GAD-7, IES-R	4,369	100	0	4,369	08/02/2020–15/02/2020

*DASS-21, depression anxiety stress scale-21; GAD-7, generalized anxiety scale-7; IES-R, impact of event scale—revised; NRS, numeric rating scale; PCL-C, the PTSD checklist—civilian version; PHQ-9, patient health questionaire-9; PQEPHE, the psychosocial questionnaire of emergency public health events; PSS, perceived stress scale; PTSD-SS, post-traumatic stress disorder self-rating scale; SAS, self-rating anxiety scale; SCL-90, symptom self-assessment scale; SDS, self-rating depression scale; SRQ-20, self-reporting questionnaire-20; SQR, stress reaction questionnaire*.

a *The study was not included in the meta-analysis*.

### Methodological Quality Assessment

All of the selected articles were assessed for methodological quality. The quality score for each study is presented in Table 2. A total of 22 studies (88.0%) were of high quality, and three studies (12.0%) were of moderate quality.

**Table 2:** Methodological quality assessment of the included studies in this systematic review.

**No**.	**First author**	**a**	**b**	**c**	**d**	**e**	**f**	**g**	**h**	**i**	**j**	**k**	**Score**	**Overall quality**
1	Zi-feng Li	Y	Y	Y	Y	Y	Y	Y	Y	N	Y	N	9	High
2	Qiu-xiang Nong	Y	N	Y	Y	Y	Y	Y	N	N	Y	N	7	Moderate
3	Yu-ning Chen	Y	N	Y	Y	Y	Y	Y	N	N	Y	N	7	Moderate
4	Na Zheng	Y	Y	Y	Y	Y	Y	Y	N	N	Y	N	8	High
5	Xiao-wen Gu	Y	N	Y	Y	Y	Y	Y	Y	N	Y	N	8	High
6	Wei	Y	Y	Y	Y	Y	Y	Y	Y	N	Y	N	9	High
7	Xiao-lei Liu	Y	Y	Y	Y	Y	Y	Y	N	N	Y	N	8	High
8	Jia-qi Xu	Y	Y	Y	Y	Y	Y	Y	Y	N	Y	N	9	High
9	Ji-zheng Huang	Y	N	Y	Y	Y	Y	Y	Y	N	Y	N	8	High
10	Xia Chen	Y	N	Y	Y	Y	Y	Y	Y	N	Y	N	8	High
11	Di Wu	Y	N	Y	Y	Y	Y	Y	Y	N	Y	N	8	High
12	Zhen-xiao Sun	Y	N	Y	Y	Y	Y	Y	Y	N	Y	N	8	High
13	Zheng Li	Y	Y	Y	Y	Y	Y	Y	Y	N	Y	N	9	High
14	Xiang-lai Liu	Y	Y	Y	Y	Y	Y	Y	Y	N	Y	N	9	High
15	Zhong-wei Guo	Y	N	Y	Y	Y	Y	Y	Y	N	Y	N	8	High
16	Xiao-ming Xu	Y	Y	Y	Y	Y	Y	Y	Y	N	Y	N	9	High
17	S. Wang	Y	N	Y	Y	Y	Y	Y	Y	N	Y	N	8	High
18	Ming-yu Si	Y	N	Y	Y	Y	Y	Y	Y	N	Y	N	8	High
19	Juan Yang	Y	N	Y	Y	Y	Y	Y	Y	N	Y	N	8	High
20	Zhong-xiang Cai	Y	Y	Y	Y	Y	Y	Y	Y	N	Y	Y	10	High
21	Teng-fei Tian	Y	N	Y	Y	Y	Y	N	Y	N	Y	N	7	Moderate
22	De-ying Hu	Y	Y	Y	Y	Y	Y	Y	Y	N	Y	N	9	High
23	Zhou Zhu	Y	Y	Y	Y	Y	Y	Y	Y	N	Y	N	9	High
24	Jian-bo Lai	Y	Y	Y	Y	Y	Y	Y	Y	N	Y	N	9	High
25	Guo Li	Y	N	Y	Y	Y	Y	Y	Y	N	Y	N	8	High

*a, define the source of information (survey, record review); b, list inclusion and exclusion criteria for exposed and unexposed subjects (cases and controls) or refer to previous publications; c, indicate the time period used for identifying patients; d, indicate whether or not the subjects were consecutive if not population-based; e, indicate if the evaluators of subjective components of study were masked to other aspects of the status of the participants; f, describe any assessments undertaken for quality assurance purposes (e.g., test/retest of primary outcome measurements); g, explain any patient exclusions from analysis; h, describe how confounding was assessed and/or controlled; i, if applicable, explain how missing data were handled in the analysis; j, summarize patient response rates and completeness of data collection; k, clarify what follow-up, if any, was expected and the percentage of patients for which incomplete data or follow-up was obtained*.

### Meta-Analysis of the Included Studies

A total of 22 studies were in the meta-analysis of pooled prevalence of anxiety disorders, and the scales used to measure anxiety included the Generalized Anxiety Scale-7 (GAD-7, *n* = 13, 59.1%), Self-rating Anxiety Scale (SAS, *n* = 7,31.8%), and Depression Anxiety Stress Scale-21 (DASS-21, *n* = 2, 9.1%).

A total of 18 studies were in the meta-analysis of pooled prevalence of depression disorders, and the scales for the depression survey included Patient Health Questionaire-9 (PHQ-9, *n* = 12, 66.7%), Self-rating Depression Scale (SDS, *n* = 4, 22.2%), and Depression Anxiety Stress Scale-21 (DASS-21, *n* = 2, 11.1%).

A total of nine studies were in the meta-analysis of pooled prevalence of stress disorders; the scales for stress disorder survey included Impact of Event Scale—Revised (IES-R, *n* = 6, 66.7%), Impact of Event Scale-6 (IES-6, *n* = 1,11.1%), the Post-traumatic Stress Disorder (PTSD) checklist—civilian version (PCL-C, *n* = 1, 11.1%), and PTSD-Rating Scale (PTSD-SS, *n* = 1, 11.1%).

**Figure 2:**
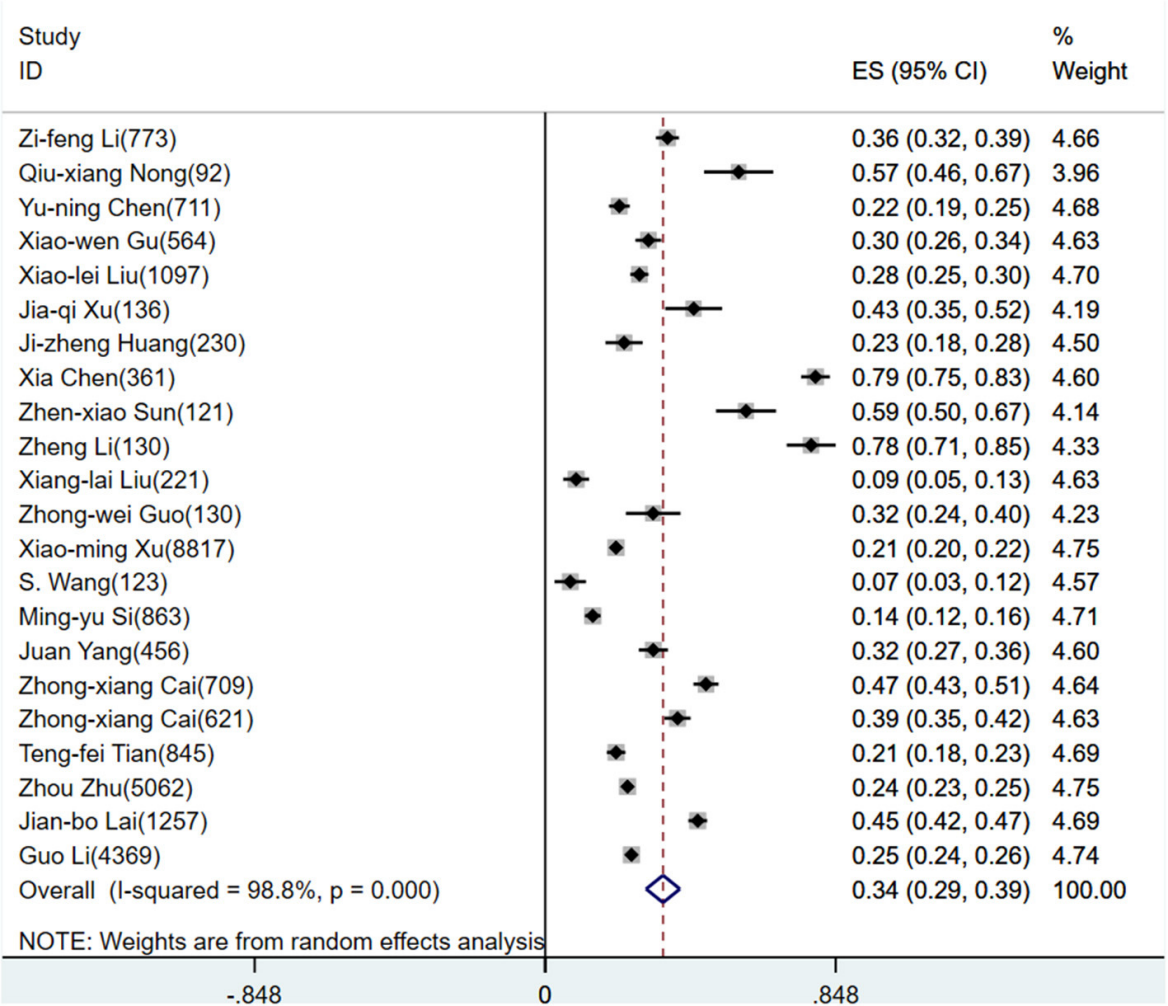
Forest plot of anxiety prevalence rate in Chinese healthcare workers.

**Figure 3:**
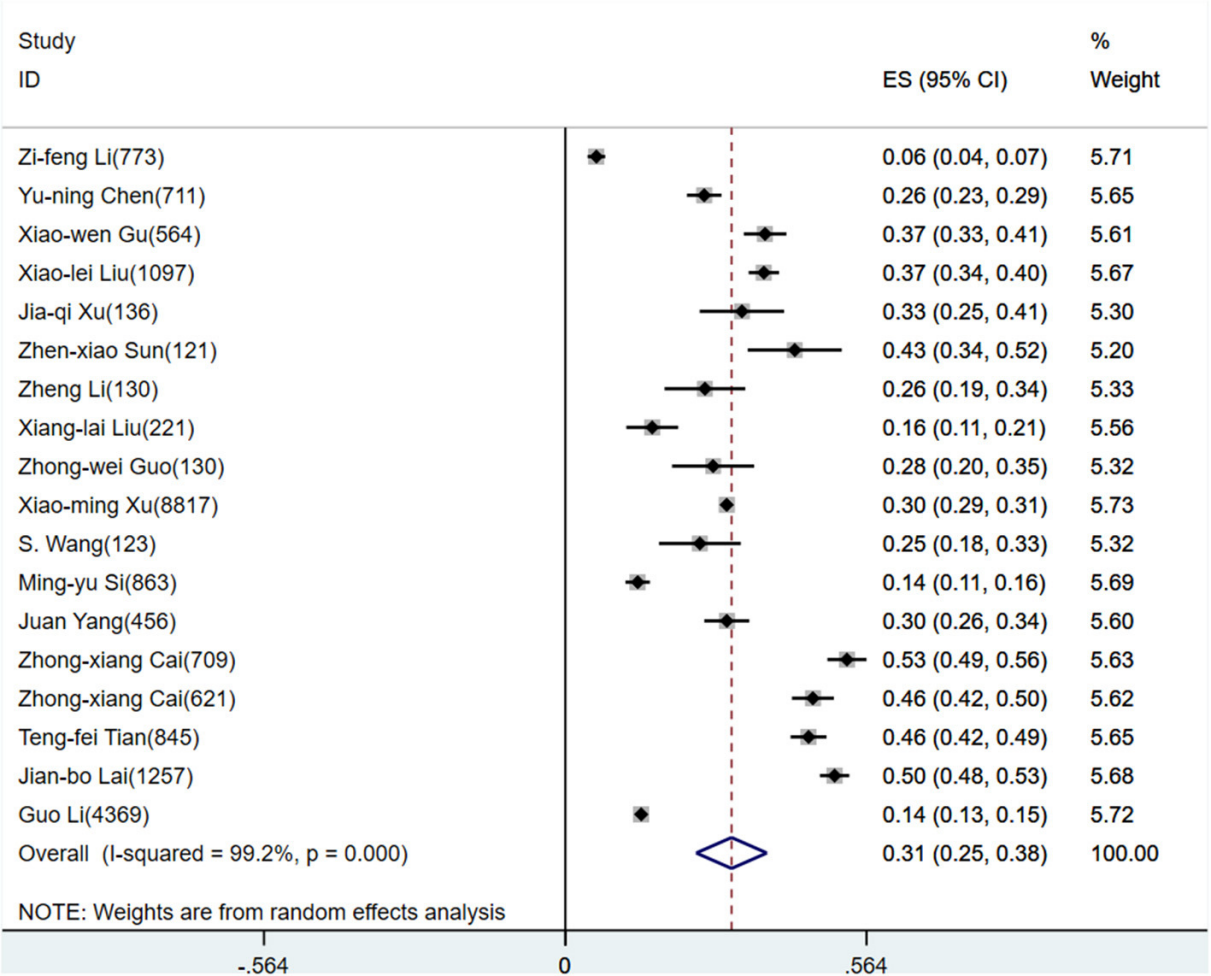
Forest plot of depression prevalence in Chinese healthcare workers.

**Figure 4:**
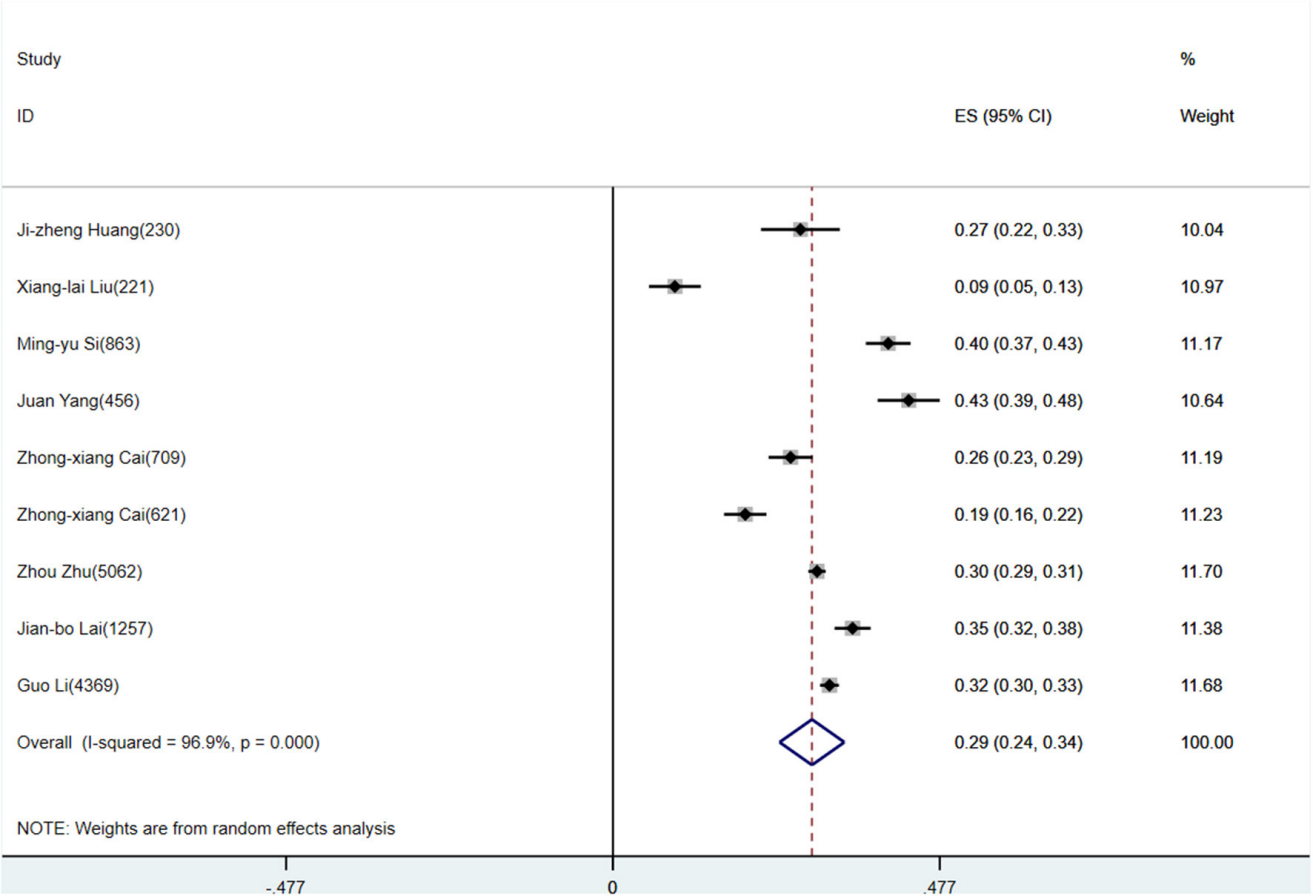
Forest plot of stress disorders prevalence in Chinese healthcare workers.

According to D+L pooled estimated value of meta-analysis of studies involving HWs with a random effects model, the prevalence of anxiety, depression, and stress disorders was 34.4% (95%CI, 29.5–39.4%), 31.1% (95%CI, 24.5–37.7%), and 29.1% (95%CI, 24.3–33.8%), respectively, for HWs. The forest plots showed a pooled prevalence of anxiety (Figure 2), depression (Figure 3), and stress disorders (Figure 4) in HWs.

Due to the need for more active treatment in people with moderate to severe anxiety or depression, we calculated the pooled prevalence of mild disorders and moderate to above separately, and the results showed that the prevalence of mild anxiety and depression disorders was 26.1% (95%CI, 20.8–31.4%) and 22.3% (95%CI, 16.6–28.0%), respectively, for HWs, and the prevalence of moderate to above anxiety and depression disorders was 10.3% (95%CI, 8.2–12.5%), 31.1% (95%CI, 24.5–37.7%), and 10.5% (95%CI, 8.0–13.0%), respectively, for HWs.

The survey time of the included studies spanned more than 3 months. According to the process of epidemic prevention and control in China, the survey time was divided into stage I (late January–early February), stage II (mid-term February), and stage III (after late February). The pooled prevalence of anxiety disorders in the three stages was stage I−46.4% (95%CI, 42.9–49.9%), stage II−28.0% (95%CI, 23.9–32.1%), and stage III−27.6% (95%CI, 16.0–39.2%), respectively. The pooled prevalence of depression disorders in the three stages was stage I−46.5% (95%CI, 38.8–54.2%), stage II−27.1% (95%CI, 19.8–34.5%), and stage III−32.9% (95%CI, 16.2–49.5%), respectively. Due to few included studies to explore stress disorders, the pooled prevalence of stress disorders in the three stages could not be divided.

### Sensitivity Analysis

Studies in the meta-analysis of anxiety and depression prevalence had considerable heterogeneity, and a sensitivity analysis was carried out to explore the origin of the heterogeneity. Subgroups of HWs were divided into frontline and overall HWs, working in Hubei Province and other regions in China, and being nurses and overall HWs. The results of the sensitivity analysis revealed that the prevalence of anxiety for frontline HWs was 34.8% (95%CI, 28.8–41.4%), higher than that for overall HWs (34.1%; 95%CI, 25.3–42.9%). The prevalence of anxiety for HWs in Hubei Province was 37.9% (95%CI, 28.7–47.1%), higher than those in other regions of China at 30.8% (95%CI, 25.1–36.5%). A subgroup of nurses was analyzed, and the results revealed that the anxiety prevalence for nurses was 44.1% (95%CI, 35.4–52.8%), significantly higher than that for overall HWs (29.0%; 95%CI, 23.4–34.7%), but the prevalence of depression for frontline HWs was 28.2% (95%CI, 18.8–37.6%), lower than that for overall HWs (34.1%; 95%CI, 24.4–43.9%). The prevalence of depression for HWs in Hubei Province was 27.5% (95%CI, 17.3–37.6%), lower than those in other regions of China at 34.7% (95%CI, 27.5–41.9%). The depression prevalence of nurses was 34.1% (95%CI, 17.5–50.7%), higher than for overall HWs (29.2%; 95%CI, 21.7–36.7%).

### Publication Bias

For studies which included data on the prevalence of anxiety, the results were as follows: Begg's test result, *z* = 0.62; *P* = 0.537; Egger's test result, *P* = 0.000. For studies with depression prevalence, the results of Begg's test was *z* = 1.29, *P* = 0.198, and that of Egger's test was *P* = 0.000. For studies which included data on the prevalence of stress disorders, the results of Begg's test was *z* = 0.52, *P* = 0.602, and that of Egger's test was *P* = 0.000. The results suggested that the funnel plot of studies which included data on prevalence was asymmetric, and there may be not a publication bias other than the reasons for the asymmetry of the funnel plot, such as studies with a small sample.

## Discussion

Our study constituted the first review of observational studies on the psychosocial impact of the COVID-19 outbreak on Chinese HWs and the changes professionals had faced due to the pandemic. The majority of population-based surveys carried out during the coronavirus pandemic have been conducted online and utilized self-assessment mental health tests. In order to obtain more accurate and representative samples, the majority of studies that we examined utilized cluster sampling. According to the survey times of the included studies, most of the research was conducted during the period when infection prevention measures were at their strictest in China. Based on the constructive information obtained for this study, our analysis aimed to guide the formulation of public health interventions designed to more effectively prevent and treat the social psychological health problems suffered by HWs resulting from the COVID-19 pandemic.

### Assessing the Mental Health of Chinese Healthcare Workers

The findings of this review reveal that nearly one-third of healthcare workers may suffer from psychosocial problems such as anxiety, depression, and stress disorders. Focusing on social psychological changes, this study selected outcome indicators with more research data in the three aspects of anxiety, depression, and stress disorder. According to existing studies, sleep disorders have also been proven to be a common psychosocial problem experienced by healthcare workers (Zhao et al., 2020). The prevalence of viral diseases and the devastating symptoms of coronavirus have had a negative impact on the mental health of healthcare workers (Salazar De Pablo et al., 2020; Serrano-Ripoll et al., 2020).

Our review showed that the Chinese HWs' psychological states varied over time. In the early stages of the pandemic, healthcare workers experienced social psychological problems that were closely related to the sudden emergence of significant stressors. Previous studies have shown that during and after the outbreak of severe acute respiratory syndrome (SARS) in China, SARS survivors, as well as the general public, experienced symptoms of anxiety, depression, and PTSD (Cheng et al., 2004; Hong et al., 2009). As the COVID-19 pandemic was brought under control and our understanding of the SARS-CoV-2 virus deepened, the mental health of Chinese HWs had seemed to have improved. However, in order to evaluate the long-term impact of this pandemic on HWs' mental health, further follow-up studies are needed.

### Factors Influencing the Mental Health of Chinese Healthcare Workers

In the results of this study, the prevalence of depression and anxiety among frontline HWs was not significantly higher than that of non-frontline HWs, indicating a need to take into account the overall social psychological status of HWs. Indeed even HWs only indirectly affected by COVID-19 are under immense pressure. The frontline HWs covered in this study included members of several national medical teams that traveled across China to Hubei Province to support the local fight against the pandemic (Li et al., 2020; Liu et al., 2020; Wu and Ding, 2020). Although some exhibited clear symptoms of anxiety, there were no clear manifestations of low motivation caused by depression, which also reflects the teams' enthusiasm to voluntarily participate in the fight against the pandemic in Hubei Province. The COVID-19 pandemic in China started in Hubei Province, the province which ultimately experienced the highest number of diagnosed patients. Therefore, the prevalence of mental health problems among HWs in Hubei Province was significantly higher than that in other regions (Cai Z. et al., 2020; Li et al., 2020).

Frontline nurses were responsible for throat swab testing as well as daily care and treatment, and therefore, they were subject to the closest contact with potential and confirmed patients of COVID-19. As such, these nurses suffered from mental health problems that were worse than those of other HWs (Li G. et al., 2020). Mobile cabin hospitals were set up mainly to treat diagnosed patients with mild symptoms and were treated as temporary treatment centers during the pandemic. As the mobile cabin hospitals quickly received a large number of diagnosed patients with mild symptoms in a short period of time, nurses became responsible for the treatment of many more patients than under normal circumstances, which likely exerted additional physical and psychological pressure on them. One study showed that nurses dispatched to mobile cabin hospitals exhibited a significantly higher risk of experiencing mental health problems than nurses in other departments (Cai Z. et al., 2020).

This review also included a survey of research on the mental health state of resident doctors (Chen Y. et al., 2020). The results revealed concerning indicators regarding the mental health of resident doctors undergoing standard training. Although these doctors were not on the frontlines diagnosing COVID-19 patients and delivering treatment, they were still involved in important medical tasks. Moreover, because they were still receiving their professional training, it is likely that the pandemic will have an impact on the future of their personal and professional development. The study results showed that the higher the current degree of the resident doctor, the higher the degree of depression and anxiety (Chen Y. et al., 2020). Accordingly, it is necessary to pay special attention to the mental health states of young healthcare workers during the professional development period.

The studies included in this review showed that psychosocial resilience and psychosocial dilemmas were closely related to social support (Gu et al., 2020; Guo et al., 2020; Li et al., 2020; Liu et al., 2020; Wu and Ding, 2020). Adequate personal protective equipment and infection prevention training for healthcare workers have a positive effect on healthcare workers' mental health (Gu et al., 2020). All these findings indicated that the mental health of HWs was affected by a variety of factors. Therefore, strategies for alleviating healthcare workers' social psychological problems must fully consider levels of social support, economic conditions, and other related factors.

### Improving the Mental Health of Healthcare Workers by Interventions

The COVID-19 pandemic has profoundly impacted all aspects of society (Holmes et al., 2020), and there is an urgent need to solve the health workers' psychosocial problems, propose potential public health interventions, and encourage people to change their lifestyles in order to improve their physical and mental health. Moreover, research on vulnerable groups must be incorporated when devising effective countermeasures. In order to avoid occupational exhaustion, a moderate level of work intensity must be maintained for both frontline and non-frontline HWs. Under the special infection prevention and control measures established during the pandemic, patients were treated in isolated spaces, especially during the early stage of the pandemic. Due to a lack of social support, more psychosocial interventions should have been provided to frontline medical staff, and remote psychological counseling services would have benefited HWs. During the pandemic, the public was required to maintain social distancing rules. Even HWs had to quarantine at home during non-working hours. During this period, workers had to maintain exercise routines and avoid focusing on negative information related to the pandemic.

Active mind–body therapies (AMBT), such as meditation, yoga, Tai Chi, and Qigong, are considered helpful practices for improving HWs' mental and physical health. Previous studies have shown that AMBT had certain therapeutic effects for those suffering from PTSD (Van der Kolk et al., 2014; Polusny et al., 2015; Duan-Porter et al., 2016; Possemato et al., 2016). Mind–body therapy can also alleviate the physical and psychological symptoms suffered by PTSD patients, allowing them to actively cope with pain and enhance their ability to practice healthy living habits (Possemato et al., 2016; Niles et al., 2018). In addition, these self-help physical and mental interventions can help HWs take their attention off stressors and initiate an active health enhancement cycle (Niles et al., 2018). Because of their low cost and simplicity, they are considered effective methods of supplementary intervention during a pandemic.

### Strengths and Limitations

This study constitutes the first systematic review of research on Chinese healthcare workers' mental health, and the study synthesized the prevalence of anxiety, depression, and stress disorders among HWs through a meta-analysis. Meta-analysis was used to explore the prevalence of psychological problems among HWs in different periods of the COVID-19 outbreak, and the research results have reflected changes in the mental states of Chinese HWs.

This review adopted relatively strict criteria of inclusion and exclusion. However, due to the characteristics of observational studies conducted during the pandemic, the studies covered in this review were significantly heterogeneous. This heterogeneity derives from the following aspects: the small sample capacity of some studies, the adoption of non-random sampling methods, and the use of survey scales with differing degrees and units of measurement. There was only one longitudinal study included in this review. Through analysis, it was found that longer-term follow-up and observation of the changes in the mental states of HWs are necessary. If possible, more longitudinal studies should be conducted. It is advisable for future longitudinal research to compare the psychosocial problems caused by the COVID-19 outbreak in different countries and regions as well as the similarities and differences in how countries coped with the effects of the outbreak. Such an approach would enable a more comprehensive understanding of the profound impact of the COVID-19 pandemic.

## Conclusion

The Chinese HWs in the COVID-19 pandemic were prone to psychosocial problems; nearly one-third of HWs had different degrees of anxiety, depression, and stress disorder. Nurses and those working in Hubei Province had a higher prevalence of anxiety and depression. More longitudinal studies should be conducted to explore the mental health of HWs in different periods of the COVID-19 pandemic. People should be able to get help to cope with the psychological impact resulting from the pandemic.

## Data Availability Statement

The raw data supporting the conclusions of this article will be made available by the authors, without undue reservation.

## Author Contributions

FD and J-pL designed the systematic review. FD and H-lL participated in searching, selecting studies, data extraction, and bias risk assessment and contributed to performing data analyses and the first draft of the manuscript. FD, H-lL, MY, C-lL, ND, YZ, NR, and J-pL were all involved in advising and critically revising the manuscript. All authors have read and approved the final manuscript.

## Conflict of Interest

The authors declare that the research was conducted in the absence of any commercial or financial relationships that could be construed as a potential conflict of interest.
