# Supplementary material for: Immediate Psychosocial Impact on Healthcare Workers During COVID-19 Pandemic in China: A Systematic Review and Meta-Analysis
Source: Front Psychol. 2021 May 28;12:645460. doi: 10.3389/fpsyg.2021.645460 (PMC8192844; doi:10.3389/fpsyg.2021.645460)
Supplement: Supplementary file 1 [file Data_Sheet_1.DOCX]

**Supplementary Materials**

**Catalogue**

[Additional file 1 PRISMA checklist of this systematic review 2](#_Toc24727)

[Additional file 2 MOOSE Checklist of this systematic review 5](#_Toc10098)

[Additional file 3 Search strategy for all databases 7](#_Toc2745)

# Additional file 1 PRISMA checklist of this systematic review

| **Section/topic** | **#** | **Checklist item** | **Reported on page #** |
| --- | --- | --- | --- |
| **TITLE** | | |  |
| Title | 1 | Identify the report as a systematic review, meta-analysis, or both. | 1 |
| **ABSTRACT** | | |  |
| Structured summary | 2 | Provide a structured summary including, as applicable: background; objectives; data sources; study eligibility criteria, participants, and interventions; study appraisal and synthesis methods; results; limitations; conclusions and implications of key findings; systematic review registration number. | 1-2 |
| **INTRODUCTION** | | |  |
| Rationale | 3 | Describe the rationale for the review in the context of what is already known. | 2-4 |
| Objectives | 4 | Provide an explicit statement of questions being addressed with reference to participants, interventions, comparisons, outcomes, and study design (PICOS). | 4 |
| **METHODS** | | |  |
| Protocol and registration | 5 | Indicate if a review protocol exists, if and where it can be accessed (e.g., Web address), and, if available, provide registration information including registration number. | 5 |
| Eligibility criteria | 6 | Specify study characteristics (e.g., PICOS, length of follow-up) and report characteristics (e.g., years considered, language, publication status) used as criteria for eligibility, giving rationale. | 4-5 |
| Information sources | 7 | Describe all information sources (e.g., databases with dates of coverage, contact with study authors to identify additional studies) in the search and date last searched. | 4-5 |
| Search | 8 | Present full electronic search strategy for at least one database, including any limits used, such that it could be repeated. | 4-5 |
| Study selection | 9 | State the process for selecting studies (i.e., screening, eligibility, included in systematic review, and, if applicable, included in the meta-analysis). | 5 |
| Data collection process | 10 | Describe method of data extraction from reports (e.g., piloted forms, independently, in duplicate) and any processes for obtaining and confirming data from investigators. | 5 |
| Data items | 11 | List and define all variables for which data were sought (e.g., PICOS, funding sources) and any assumptions and simplifications made. | 5 |
| Risk of bias in individual studies | 12 | Describe methods used for assessing risk of bias of individual studies (including specification of whether this was done at the study or outcome level), and how this information is to be used in any data synthesis. | 5 |
| Summary measures | 13 | State the principal summary measures (e.g., risk ratio, difference in means). | 5-6 |
| Synthesis of results | 14 | Describe the methods of handling data and combining results of studies, if done, including measures of consistency (e.g., I^2^) for each meta-analysis. | 5-6 |
| Risk of bias across studies | 15 | Specify any assessment of risk of bias that may affect the cumulative evidence (e.g., publication bias, selective reporting within studies). | 5-6 |
| Additional analyses | 16 | Describe methods of additional analyses (e.g., sensitivity or subgroup analyses, meta-regression), if done, indicating which were pre-specified. | 6 |
| **RESULTS** | | |  |
| Study selection | 17 | Give numbers of studies screened, assessed for eligibility, and included in the review, with reasons for exclusions at each stage, ideally with a flow diagram. | 6 |
| Study characteristics | 18 | For each study, present characteristics for which data were extracted (e.g., study size, PICOS, follow-up period) and provide the citations. | 6 |
| Risk of bias within studies | 19 | Present data on risk of bias of each study and, if available, any outcome level assessment (see item 12). | 6 |
| Results of individual studies | 20 | For all outcomes considered (benefits or harms), present, for each study: (a) simple summary data for each intervention group (b) effect estimates and confidence intervals, ideally with a forest plot. | 7 |
| Synthesis of results | 21 | Present results of each meta-analysis done, including confidence intervals and measures of consistency. | 7 |
| Risk of bias across studies | 22 | Present results of any assessment of risk of bias across studies (see Item 15). | 7-8 |
| Additional analysis | 23 | Give results of additional analyses, if done (e.g., sensitivity or subgroup analyses, meta-regression [see Item 16]). | 7-8 |
| DISCUSSION | | |  |
| Summary of evidence | 24 | Summarize the main findings including the strength of evidence for each main outcome; consider their relevance to key groups (e.g., healthcare providers, users, and policy makers). | 8-10 |
| Limitations | 25 | Discuss limitations at study and outcome level (e.g., risk of bias), and at review-level (e.g., incomplete retrieval of identified research, reporting bias). | 10 |
| Conclusions | 26 | Provide a general interpretation of the results in the context of other evidence, and implications for future research. | 10 |
| FUNDING | | |  |
| Funding | 27 | Describe sources of funding for the systematic review and other support (e.g., supply of data); role of funders for the systematic review. | 11 |

# Additional file 2 MOOSE Checklist of this systematic review

| **Item No** | **Recommendation** | **Reported on Page No** |
| --- | --- | --- |
| Reporting of background should include | | |
| 1 | Problem definition | 2-3 |
| 2 | Hypothesis statement | 3 |
| 3 | Description of study outcome(s) | 3 |
| 4 | Type of exposure or intervention used | 3 |
| 5 | Type of study designs used | 4 |
| 6 | Study population | 4 |
| Reporting of search strategy should include | | |
| 7 | Qualifications of searchers (eg, librarians and investigators) | 4 |
| 8 | Search strategy, including time period included in the synthesis and key words | 4 |
| 9 | Effort to include all available studies, including contact with authors | 5 |
| 10 | Databases and registries searched | 4 |
| 11 | Search software used, name and version, including special features used (eg, explosion) | 4 |
| 12 | Use of hand searching (eg, reference lists of obtained articles) | 5 |
| 13 | List of citations located and those excluded, including justification | 4-5 |
| 14 | Method of addressing articles published in languages other than English | 4 |
| 15 | Method of handling abstracts and unpublished studies | 4 |
| 16 | Description of any contact with authors | 4 |
| Reporting of methods should include | | |
| 17 | Description of relevance or appropriateness of studies assembled for assessing the hypothesis to be tested | 5 |
| 18 | Rationale for the selection and coding of data (eg, sound clinical principles or convenience) | 5 |
| 19 | Documentation of how data were classified and coded (eg, multiple raters, blinding and interrater reliability) | 5 |
| 20 | Assessment of confounding (eg, comparability of cases and controls in studies where appropriate) | 5 |
| 21 | Assessment of study quality, including blinding of quality assessors, stratification or regression on possible predictors of study results | 5 |
| 22 | Assessment of heterogeneity | 6 |
| 23 | Description of statistical methods (eg, complete description of fixed or random effects models, justification of whether the chosen models account for predictors of study results, dose-response models, or cumulative meta-analysis) in sufficient detail to be replicated | 6 |
| 24 | Provision of appropriate tables and graphics | 6 |
| Reporting of results should include | | |
| 25 | Graphic summarizing individual study estimates and overall estimate | 6 |
| 26 | Table giving descriptive information for each study included | 6 |
| 27 | Results of sensitivity testing (eg, subgroup analysis) | 7-8 |
| 28 | Indication of statistical uncertainty of findings | 7-8 |

| Reporting of discussion should include | | |
| --- | --- | --- |
| 29 | Quantitative assessment of bias (eg, publication bias) | 8,10 |
| 30 | Justification for exclusion (eg, exclusion of non-English language citations) | 10 |
| 31 | Assessment of quality of included studies | 10 |
| Reporting of conclusions should include | | |
| 32 | Consideration of alternative explanations for observed results | 10 |
| 33 | Generalization of the conclusions (ie, appropriate for the data presented and within the domain of the literature review) | 10 |
| 34 | Guidelines for future research | 10 |
| 35 | Disclosure of funding source | 11 |

*From*: Stroup DF, Berlin JA, Morton SC, et al, for the Meta-analysis Of Observational Studies in Epidemiology (MOOSE) Group. Meta-analysis of Observational Studies in Epidemiology. A Proposal for Reporting. *JAMA*. 2000;283(15):2008-2012. doi: 10.1001/jama.283.15.2008.

Transcribed from the original paper within the NEUROSURGERY® Editorial Office, Atlanta, GA, United Sates. August 2012.

# Additional file 3 Search strategy for all databases

**1.Pubmed**

(((NCP[Title/Abstract] OR NCIP[Title/Abstract] OR 2019-nCOV[Title/Abstract] OR COVID-19[Title/Abstract] OR novel coronavirus[Title/Abstract] OR 2019 novel coronavirus[Title/Abstract] OR coronavirus 2019[Title/Abstract] OR coronavirus-19[Title/Abstract] OR corona virus disease-19[Title/Abstract] OR coronavirus disease 2019[Title/Abstract] OR corona virus disease 2019[Title/Abstract] OR coronavirus disease-19[Title/Abstract] OR novel coronavirus pneumonia[Title/Abstract] OR SARS-CoV-2[Title/Abstract] OR Wuhan pneumonia[Title/Abstract] OR new coronavirus[Title/Abstract] OR pneumonia caused by the novel coronavirus[Title/Abstract] OR novel coronavirus-caused pneumonia[Title/Abstract] OR novel coronavirus-infected pneumonia[Title/Abstract] OR Severe acute respiratory syndrome coronavirus 2[Title/Abstract] OR novel coronavirus 2019-nCoV infected pneumonia ncip title/abstract OR novel coronavirus pneumonia[Title/Abstract] OR 2019 novel corona virus[Title/Abstract] OR COVID19[Title/Abstract] OR Coronavirus Infections[Title/Abstract] OR COVID Infection[Title/Abstract] OR 2019 novel coronavirus disease[Title/Abstract] OR 2019 novel coronavirus infection disease[Title/Abstract]) AND (psycho*[Title/Abstract] OR depress*[Title/Abstract] OR mental*[Title/Abstract] OR anxiety[Title/Abstract] OR stress*[Title/Abstract] OR acute stress disorder [Title/Abstract] OR post-traumatic stress disorder[Title/Abstract] OR sleep[Title/Abstract] OR somatization[Title/Abstract] OR exhaust*[Title/Abstract] OR psychological assistance[Title/Abstract] OR spirit*[Title/Abstract] OR trauma*[Title/Abstract] OR social support[Title/Abstract] OR stigma[Title/Abstract] OR psychological crisis[Title/Abstract] OR psychological experience[Title/Abstract] OR insomnia[Title/Abstract] OR ASD[Title/Abstract] OR PTSD[Title/Abstract])) ) AND (("2020/01/01"[Date - Publication] : "2020/10/07"[Date - Publication]))

**2.Embase**

(psycho*:ti,ab,kw OR mental*:ti,ab,kw OR depress*:ti,ab,kw OR anxiety:ti,ab,kw OR stress*:ti,ab,kw OR 'acute stress disorder':ti,ab,kw OR 'post-traumatic stress disorder':ti,ab,kw OR sleep:ti,ab,kw OR somatization:ti,ab,kw OR exhaust:ti,ab,kw OR 'psychological assistance':ti,ab,kw OR spirit*:ti,ab,kw OR trauma*:ti,ab,kw OR 'social support':ti,ab,kw OR stigma:ti,ab,kw OR 'psychological crisis':ti,ab,kw OR 'psychological experience':ti,ab,kw OR insomnia:ti,ab,kw OR asd:ti,ab,kw OR ptsd:ti,ab,kw) AND (ncp:ti,ab,kw OR ncip:ti,ab,kw OR '2019 ncov':ti,ab,kw OR 'covid 19':ti,ab,kw OR 'novel coronavirus':ti,ab,kw OR '2019 novel coronavirus':ti,ab,kw OR 'coronavirus 2019':ti,ab,kw OR 'coronavirus disease-19':ti,ab,kw OR 'coronavirus disease 19':ti,ab,kw OR 'corona virus disease 19':ti,ab,kw OR 'sars cov 2':ti,ab,kw OR 'wuhan pneumonia':ti,ab,kw OR 'new coronavirus':ti,ab,kw OR 'pneumonia caused by the novel coronavirus':ti,ab,kw OR 'novel coronavirus-caused pneumonia':ti,ab,kw OR 'novel coronavirus-infected pneumonia':ti,ab,kw OR 'severe acute respiratory syndrome coronavirus 2':ti,ab,kw OR 'novel coronavirus 2019-ncov-infected pneumonia ncip':ti,ab,kw OR 'novel coronavirus pneumonia':ti,ab,kw OR 'Coronavirus Infections':ti,ab,kw OR 'COVID Infection':ti,ab,kw OR '2019 novel coronavirus disease':ti,ab,kw OR '2019 novel coronavirus infection disease':ti,ab,kw) 2020-2021

**3.PscyINFO**

#1. (psycho* or mental* or depress* or anxiety or stress* or acute stress disorder or post-traumatic stress disorder or sleep or somatization or exhaust* or psychological assistance or spirit* or trauma* or social support or stigma or psychological crisis or psychological experience or insomnia or ASD or PTSD).ti. or (psycho* or mental* or depress* or anxiety or stress* or acute stress disorder or post-traumatic stress disorder or sleep or somatization or exhaust* or psychological assistance or spirit* or trauma* or social support or stigma or psychological crisis or psychological experience or insomnia or ASD or PTSD).ab. or (psycho* or mental* or depress* or anxiety or stress* or acute stress disorder or post-traumatic stress disorder or sleep or somatization or exhaust* or psychological assistance or spirit* or trauma* or social support or stigma or psychological crisis or psychological experience or insomnia or ASD or PTSD).id.2019-nCoV. ab,hw,mh,ti. OR SARS-CoV-2. ab,hw,mh,ti. OR coronavirus. ab,hw,mh,ti. OR COVID-19. ab,hw,mh,ti. OR new coronavirus. ab,hw,mh,ti. OR nCoV. ab,hw,mh,ti. OR novel coronavirus. ab,hw,mh,ti.

#2. (NCP or NCIP or 2019-nCOV or COVID-19 or novel coronavirus or 2019 novel coronavirus or coronavirus 2019 or coronavirus-19 or corona virus disease-19 or coronavirus disease 2019 or corona virus disease 2019 or coronavirus disease-19 or novel coronavirus pneumonia or SARS-CoV-2 or Wuhan pneumonia or new coronavirus or pneumonia caused by the novel coronavirus or novel coronavirus-caused pneumonia or novel coronavirus-infected pneumonia or Severe acute respiratory syndrome coronavirus 2 or novel coronavirus 2019-nCoV infected pneumonia ncip title abstract or novel coronavirus pneumonia or 2019 novel corona virus or COVID19 or Coronavirus Infections or COVID Infection or 2019 novel coronavirus disease or 2019 novel coronavirus infection disease).ti. or (NCP or NCIP or 2019-nCOV or COVID-19 or novel coronavirus or 2019 novel coronavirus or coronavirus 2019 or coronavirus-19 or corona virus disease-19 or coronavirus disease 2019 or corona virus disease 2019 or coronavirus disease-19 or novel coronavirus pneumonia or SARS-CoV-2 or Wuhan pneumonia or new coronavirus or pneumonia caused by the novel coronavirus or novel coronavirus-caused pneumonia or novel coronavirus-infected pneumonia or Severe acute respiratory syndrome coronavirus 2 or novel coronavirus 2019-nCoV infected pneumonia ncip title abstract or novel coronavirus pneumonia or 2019 novel corona virus or COVID19 or Coronavirus Infections or COVID Infection or 2019 novel coronavirus disease or 2019 novel coronavirus infection disease).ab. or (NCP or NCIP or 2019-nCOV or COVID-19 or novel coronavirus or 2019 novel coronavirus or coronavirus 2019 or coronavirus-19 or corona virus disease-19 or coronavirus disease 2019 or corona virus disease 2019 or coronavirus disease-19 or novel coronavirus pneumonia or SARS-CoV-2 or Wuhan pneumonia or new coronavirus or pneumonia caused by the novel coronavirus or novel coronavirus-caused pneumonia or novel coronavirus-infected pneumonia or Severe acute respiratory syndrome coronavirus 2 or novel coronavirus 2019-nCoV infected pneumonia ncip title abstract or novel coronavirus pneumonia or 2019 novel corona virus or COVID19 or Coronavirus Infections or COVID Infection or 2019 novel coronavirus disease or 2019 novel coronavirus infection disease).id.

#3. limit 1 to yr="2020 - 2021"

#4. #1 AND #2 AND #3

1. **CNKI (Chinese database)**

(TI = '心理' + '情绪' + '心身' + '抑郁' + '焦虑' + '应激' + '急性应激障碍' + '创伤后应激障碍' + '压力' + '睡眠' + '躯体化' + '耗竭' + '心理援助' + '精神' + '社会支持' + '病耻感' + '心理危机' + '心理体验' + '失眠' + '创伤' OR AB = '心理' + '情绪' + '心身' + '抑郁' + '焦虑' + '应激' + '急性应激障碍' + '创伤后应激障碍' + '压力' + '睡眠' + '躯体化' + '耗竭' + '心理援助' + '精神' + '社会支持' + '病耻感' + '心理危机' + '心理体验'+ '失眠' + '创伤'OR KY = '心理' + '情绪' + '心身' + '抑郁' + '焦虑' + '应激' + '急性应激障碍' + '创伤后应激障碍' + '压力' + '睡眠' + '躯体化' + '耗竭' + '心理援助' + '精神' + '社会支持' + '病耻感' + '心理危机' + '心理体验'+ '失眠' + '创伤') AND (TI = '新冠' + '新冠肺炎' + '新冠病毒' + '武汉肺炎' + '新型冠状病毒' + '2019冠状病毒' + '新型冠状病毒肺炎' + '武汉新型冠状病毒' + '新型冠状病毒感染肺炎' + '2019新型冠状病毒' + '冠状病毒肺炎' + '冠状病毒感染' + '新冠病毒肺炎' + '2019新型冠状病毒病' + '2019冠状病毒病' + '严重急性呼吸综合征冠状病毒2' + 'COVID-19' + 'SARS-CoV-2' + '2019-nCoV' + 'NCP' + '2019 新型冠状病毒肺炎' + '2019冠状病毒疾病' + '2019 新型冠状病毒肺炎' + '2019 新型冠状病毒肺炎(COVID 19)'+ 'SARS nCoV' + 'COVD1 19' + 'COVID 19肺炎' + '不明原因肺炎' + '病毒性肺炎' + '感染性肺炎' + '严重急性呼吸道综合征冠状病毒2型' + '新型冠状病毒(COVID 19)' + '新冠肺炎疫情' + '新型冠状病毒2(SARS CoV 2)' + '新型冠状病毒病' + '新型冠状病毒疾病' + '新冠状病毒' + '新型冠状病毒性肺炎' + '肺炎病毒' + '人冠状病毒' + '冠状病毒病' + '冠状病毒疾病 19' + '冠状病毒感染疾病 19' + '第二株SARS 冠状病毒' OR AB = '新冠' + '新冠肺炎' + '新冠病毒' + '武汉肺炎' + '新型冠状病毒' + '2019冠状病毒' + '新型冠状病毒肺炎' + '武汉新型冠状病毒' + '新型冠状病毒感染肺炎' + '2019新型冠状病毒' + '冠状病毒肺炎' + '冠状病毒感染' + '新冠病毒肺炎' + '2019新型冠状病毒病' + '2019冠状病毒病' + '严重急性呼吸综合征冠状病毒2' + 'COVID-19' + 'SARS-CoV-2' + '2019-nCoV' + 'NCP' + '2019 新型冠状病毒肺炎' + '2019冠状病毒疾病' + '2019 新型冠状病毒肺炎' + '2019 新型冠状病毒肺炎(COVID 19)'+ 'SARS nCoV' + 'COVD1 19' + 'COVID 19肺炎' + '不明原因肺炎' + '病毒性肺炎' + '感染性肺炎' + '严重急性呼吸道综合征冠状病毒2型' + '新型冠状病毒(COVID 19)' + '新冠肺炎疫情' + '新型冠状病毒2(SARS CoV 2)' + '新型冠状病毒病' + '新型冠状病毒疾病' + '新冠状病毒' + '新型冠状病毒性肺炎' + '肺炎病毒' + '人冠状病毒' + '冠状病毒病' + '冠状病毒疾病 19' + '冠状病毒感染疾病 19' + '第二株SARS 冠状病毒' OR KY = '新冠' + '新冠肺炎' + '新冠病毒' + '武汉肺炎' + '新型冠状病毒' + '2019冠状病毒' + '新型冠状病毒肺炎' + '武汉新型冠状病毒' + '新型冠状病毒感染肺炎' + '2019新型冠状病毒' + '冠状病毒肺炎' + '冠状病毒感染' + '新冠病毒肺炎' + '2019新型冠状病毒病' + '2019冠状病毒病' + '严重急性呼吸综合征冠状病毒2' + 'COVID-19' + 'SARS-CoV-2' + '2019-nCoV' + 'NCP' + '2019 新型冠状病毒肺炎' + '2019冠状病毒疾病' + '2019 新型冠状病毒肺炎' + '2019 新型冠状病毒肺炎(COVID 19)'+ 'SARS nCoV' + 'COVD1 19' + 'COVID 19肺炎' + '不明原因肺炎' + '病毒性肺炎' + '感染性肺炎' + '严重急性呼吸道综合征冠状病毒2型' + '新型冠状病毒(COVID 19)' + '新冠肺炎疫情' + '新型冠状病毒2(SARS CoV 2)' + '新型冠状病毒病' + '新型冠状病毒疾病' + '新冠状病毒' + '新型冠状病毒性肺炎' + '肺炎病毒' + '人冠状病毒' + '冠状病毒病' + '冠状病毒疾病 19' + '冠状病毒感染疾病 19' + '第二株SARS 冠状病毒') From 2020/01/01 to 2020/10/07

限定文献分类：医药卫生科技+心理学

1. **Wangfang (Chinese database)**

主题:("心理"+"情绪"+"心身"+"抑郁"+"焦虑"+"应激"+"急性应激障碍"+"创伤后应激障碍"+"压力"+"睡眠"+"躯体化"+"耗竭"+"心理援助"+"精神"+"创伤"+"社会支持"+"病耻感"+"心理危机"+"心理体验"+"失眠") and ("新冠"+"新冠肺炎"+"新冠病毒"+"武汉肺炎"+"新型冠状病毒"+"2019冠状病毒"+"新型冠状病毒肺炎"+"武汉新型冠状病毒"+"新型冠状病毒感染肺炎"+"2019新型冠状病毒"+"冠状病毒肺炎"+"冠状病毒感染"+"新冠病毒肺炎"+"2019新型冠状病毒病"+"2019冠状病毒病"+"严重急性呼吸综合征冠状病毒2"+"COVID-19"+"SARS-CoV-2"+"2019-nCoV"+"NCP"+"2019 新型冠状病毒肺炎"+"2019冠状病毒疾病"+"2019 新型冠状病毒肺炎"+"2019 新型冠状病毒肺炎(COVID 19)"+"SARS nCoV"+"COVD1 19"+"COVID 19肺炎"+"不明原因肺炎"+"病毒性肺炎"+"感染性肺炎"+"严重急性呼吸道综合征冠状病毒2型"+"新型冠状病毒(COVID 19)"+"新冠肺炎疫情"+"新型冠状病毒2(SARS CoV 2)"+"新型冠状病毒病"+"新型冠状病毒疾病"+"新冠状病毒"+"新型冠状病毒性肺炎"+"肺炎病毒"+"人冠状病毒"+"冠状病毒病"+"冠状病毒疾病 19"+"冠状病毒感染疾病 19"+"第二株SARS 冠状病毒")*Date:2020-2020

1. **VIP (Chinese database)**

#1 文摘=心理+情绪+心身+抑郁+焦虑+应激+压力+睡眠+躯体化+心理援助+精神+病耻感+失眠

#2 题名或关键词=心理+情绪+心身+抑郁+焦虑+应激+压力+睡眠+躯体化+心理援助+精神+病耻感+失眠

#3 #1 or #2

#4 文摘=新冠+新型冠状病毒+"2019冠状病毒"+武汉肺炎+冠状病毒肺炎+新冠病毒肺炎+新冠病毒+新冠肺炎+"COVID-19"+"SARS-CoV-2"+"2019-nCoV"+NCP

#5 题名或关键词=新冠+新型冠状病毒+"2019冠状病毒"+武汉肺炎+冠状病毒肺炎+新冠病毒肺炎+新冠病毒+新冠肺炎+"COVID-19"+"SARS-CoV-2"+"2019-nCoV"+NCP

#6 #4 or #5

#7 years 2020 to 2020

#8 学科：医药卫生

#9 #3 and #6 and #7 and #8

1. **Sinomed (Chinese database)**

检索条件 : ("心理"[常用字段:智能] OR "情绪"[常用字段:智能] OR "心身"[常用字段:智能] OR "抑郁"[常用字段:智能] OR "焦虑"[常用字段:智能] OR "应激"[常用字段:智能] OR "急性应激障碍"[常用字段:智能] OR "创伤后应激障碍"[常用字段:智能] OR "压力"[常用字段:智能] OR "睡眠"[常用字段:智能] OR "躯体化"[常用字段:智能] OR "耗竭"[常用字段:智能] OR "心理援助"[常用字段:智能] OR "精神"[常用字段:智能] OR "创伤"[常用字段:智能] OR "社会支持"[常用字段:智能] OR "病耻感"[常用字段:智能] OR "心理危机"[常用字段:智能] OR "心理体验"[常用字段:智能] OR "失眠"[常用字段:智能]) AND ("2019 新型冠状病毒肺炎"[常用字段:智能] OR "2019冠状病毒疾病"[常用字段:智能] OR "2019 新型冠状病毒肺炎(COVID 19)"[常用字段:智能] OR "SARS nCoV"[常用字段:智能] OR "COVD1 19"[常用字段:智能] OR "COVID 19肺炎"[常用字段:智能] OR "不明原因肺炎"[常用字段:智能] OR "病毒性肺炎"[常用字段:智能] OR "感染性肺炎"[常用字段:智能] OR "严重急性呼吸道综合征冠状病毒2型"[常用字段:智能] OR "新型冠状病毒(COVID 19)"[常用字段:智能] OR "新冠肺炎疫情"[常用字段:智能] OR "新型冠状病毒2(SARS CoV 2)"[常用字段:智能] OR "新型冠状病毒病"[常用字段:智能] OR "新型冠状病毒疾病"[常用字段:智能] OR "新冠状病毒"[常用字段:智能] OR "新型冠状病毒性肺炎"[常用字段:智能] OR "肺炎病毒"[常用字段:智能] OR "人冠状病毒"[常用字段:智能] OR "冠状病毒病"[常用字段:智能] OR "冠状病毒疾病 19"[常用字段:智能] OR "冠状病毒感染疾病 19"[常用字段:智能] OR "第二株SARS 冠状病毒"[常用字段:智能] OR "新冠"[常用字段:智能] OR "新冠肺炎"[常用字段:智能] OR "新冠病毒"[常用字段:智能] OR "武汉肺炎"[常用字段:智能] OR "新型冠状病毒"[常用字段:智能] OR "2019冠状病毒"[常用字段:智能] OR "新型冠状病毒肺炎"[常用字段:智能] OR "武汉新型冠状病毒"[常用字段:智能] OR "新型冠状病毒感染肺炎"[常用字段:智能] OR "2019新型冠状病毒"[常用字段:智能] OR "冠状病毒肺炎"[常用字段:智能] OR "冠状病毒感染"[常用字段:智能] OR "新冠病毒肺炎"[常用字段:智能] OR "2019新型冠状病毒病"[常用字段:智能] OR "2019冠状病毒病"[常用字段:智能] OR "严重急性呼吸综合征冠状病毒2"[常用字段:智能] OR "COVID-19"[常用字段:智能] OR "SARA-CoV-2"[常用字段:智能] OR "2019-nCoV"[常用字段:智能] OR "NCP"[常用字段:智能])

Limited 2020-2020
